# Effects of Acupuncture Knife on Inflammatory Factors and Pain in Third Lumbar Vertebrae Transverse Process Syndrome Model Rats

**DOI:** 10.1155/2014/892406

**Published:** 2014-12-02

**Authors:** Jia Ni Yu, Chang Qing Guo, Bo Hu, Nai Gang Liu, Hong Mei Sun, Hong Xu, Hai Xia Wu, Yan Guo, Chu Xi Liang, Zhan Xia Chen, Xiao Hong Li

**Affiliations:** ^1^School of Acupuncture and Moxibustion, Beijing University of Chinese Medicine, No. 11 North 3rd Ring East Road, Chaoyang District, Beijing 100029, China; ^2^Acupuncture & Moxibustion and Physiotherapy Department, Beijing Shuili Hospital, No. 19 Yu Yuan Tan South Road, Haidian District, Beijing 100036, China; ^3^Acupuncture and Moxibustion Department, China-Japan Friendship Hospital, No. 2 The Ying Hua Yuan East Street, Chaoyang District, Beijing 100029, China; ^4^School of Basic Medical Sciences, Beijing University of Chinese Medicine, No. 11 North 3rd Ring East Road, Chaoyang District, Beijing 100029, China

## Abstract

The aim of this paper was to explore the long-term effects and pain relief mechanism of acupuncture knife on third lumbar vertebrae (L_3_) transverse process syndrome. Forty SD rats were randomized into control, model, electroacupuncture (EA), and acupuncture knife (AK) group. Except control rats, other rats were subjected to an operation to emulate L_3_ transverse process syndrome. Fourteen days after the operation, EA and AK rats were given electroacupuncture and acupuncture knife treatments, respectively. Fifty-six days after the operation, enzyme-linked immunosorbent assay was used to measure substance P (SP), 5-hydroxytryptamine (5-HT), interleukin-1*β* (IL-1*β*), interleukin-10 (IL-10), tumor necrosis factor-*α* (TNF-*α*), and transforming growth factor-*β* (TGF-*β*) in peripheral blood. The tail flick test was used to observe pain threshold. We found that rats with the simulation operation had significantly higher levels of SP, 5-HT, IL-1, IL-10, TNF-*α*, and TGF-*β*, while the AK rats had lower levels. In addition, the pain threshold of AK rats was similar to that of control rats. AK pretreatment could alleviate pain through modulating inflammatory response.

## 1. Introduction

Low back pain is one of the most common medical problems and is caused by myofascial, intervertebral disk (IVD), facet joint, or sacroiliac joint problems. The third lumbar vertebra (L_3_), which adheres to most muscles, fascia, and other soft tissues of lumbar, is the only vertebra without protection of the costal bone or iliac bone. However, L_3_ is located in the most prominent part in the lumbar spine [1]. L_3_ transverse process syndrome is characterized by regional tenderness caused by overuse and injury of soft tissues adhering to the transverse process of L_3_. This syndrome is caused by peripheral sensitization of local muscle nociceptors.

Acupuncture knife, invented by Professor Hanzhang Zhu, is a novel therapy combining traditional Chinese medicine meridian theory with modern surgical techniques that uses a needle-knife as a therapeutic tool. According to previous randomized controlled multicenter clinical study, acupuncture knife can restore the dynamic balance and relieve pain by relaxing and stripping the taut node and cord formed in the L_3_ transverse process tip, which is more effective than that of other medical produces [2, 3]. Our early stage studies showed that acupuncture knife can adjust analgesic compounds in the short-term [4, 5]. However, there is no preclinical research about the mechanisms of acupuncture knife on long-term analgesia. Substance P (SP) and 5-hydroxytryptamine (5-HT) in the central nervous system and in the periphery have long been considered to have an important role in the control of pain [6, 7]. In addition, animal studies have shown that inflammatory cytokines contribute to the development of hyperalgesia [8].

In the present study, we aimed to understand the mechanism of acupuncture knife on third lumbar vertebrae transverse process syndrome and its effect on substance P (SP), 5-hydroxytryptamine (5-HT), interleukin-1*β* (IL-1*β*), interleukin-10 (IL-10), tumor necrosis factor-*α* (TNF-*α*), and transforming growth factor-*β* (TGF-*β*) in blood serum.

## 2. Materials and Methods

### 2.1. Animals and Surgical Protocol

Forty 3-month-old male Sprague-Dawley (SD) rats were used in this study, weighing 250–270 g. All rats were purchased from the Victoria-Lihua Animal Laboratory Center with batch number SCXK (Beijing, China) 2007-0001.

The rats were housed in a controlled environment (12 h light/dark cycle, room temperature 23 ± 2°C, and 50–60% relative humidity), with food and water provided according to the requirements of the Provision and General Recommendations of National Institutes of Health Guide for the Care and Use of Laboratory Animals. The Animal Research Ethics Board of Beijing University of Chinese Medicine approved the experiment.

Rats were randomly divided into four groups (*n* = 10): a control group, in which rats were not given intervention; a model group, in which rats received surgery to simulate L_3_ transverse process syndrome without treatment; an electroacupuncture (EA) group, in which rats received surgery and electroacupuncture treatment 14 days after surgery; and an acupuncture knife (AK) group, in which rats received surgery and acupuncture knife treatment 14 days after surgery. The method of establishing L_3_ transverse process syndrome model was modified from that of Wang et al. [9]. Before the surgery, the rats were fixed in a prone position with 10% chloral hydrate (Beijing Factory of Chemical Reagents, Beijing, China) abdominal anesthetization (0.4 g/kg). The hair around the L_3_ transverse process tip was removed and a 1 cm vertical incision was made 0.5–0.8 cm to the left of the L_3_-L_4_ spinal transverse process under aseptic condition. The deep myofascia was separated and the left lumbar paraspinal muscle was exposed 0.5–0.8 cm left of the central line. The paraspinal muscle was separated to the posterior of L_3_ transverse process, and a piece of 0.5 × 0.5 cm absorbent gelatin sponge was implanted (Nanjing Jinling Pharmaceutical Co., Ltd., Nanjing, China). After surgery, the lumbar paraspinal muscle of rats was sutured with plain gut suture (3-0) and the cutaneous incision with silk (4-0). The wound was sterilized with gentamicin (2 mL, 80,000 U, Tianjin Pharmaceutical Co., Ltd., Tianjin, China) to avoid infection.

### 2.2. Treatment and Techniques

For the EA group (*n* = 10), 14 days after creating the model, intermittent direct EA was performed every other day six times in 2 weeks, 20 min each time, with a frequency of 100 Hz dense-disperse wave and 2 mA current. EA was conducted with the rats lightly anaesthetized with diethyl ether for about 1 min until all four limbs were restrained. Two points were chosen: Yaoyangguan (GV 3) and left Shenshu (BL 23). Disposable, stainless sterile handle needles (Beijing Zhongyan Taihe Medical Instrument Co., Ltd., Tianjin, China) with a length of 30 mm and a diameter of 0.30 mm were used. The rats were fixed in the prone position and the acupuncture points were shaved and disinfected with 75% alcohol. Both needles were inserted percutaneously at the tip of the acupuncture points. When the acupuncturist felt the deqi sensation of the subject, the anode lead of the electric stimulator (Han's 200E, Nanjing Jisheng Medical Co. Ltd., Jiangsu, China) was connected with BL 23, and the cathode lead was connected with GV 3.

For the AK group (*n* = 10), 14 days after creating the model, acupuncture knife treatment was performed every 7 days in 2 weeks, two times in total. AK was conducted with the rats lightly anaesthetized with diethyl ether for about 1 min until all four limbs were restrained. Pathological nodes or cords as acupuncture knife points, which were much firmer than the surrounding muscle fibers, were found by gently palpated. After acupuncture knife points were shaved and disinfected with 75% alcohol. The instrument was used to make three cuts parallel to the spine and was then rotated 90° to make another cut. The instrument was removed and the wound was compressed with gauze to avoid excessive bleeding.

After treatments were finished, all rats were fed for another 28 days without any treatments or interventions.

### 2.3. Assessment

#### 2.3.1. Pain Threshold

At selected time points (all before noon), day 0 (the day before model establishment), day 2, day 8, day 14 (the day before performing treatment), day 17 (1 day after the first acupuncture knife treatment), day 21, day 28 (2 days after treatment), day 35, day 42, day 49, and day 56, the tail flick test was conducted with a noxious thermal stimulation focus on the site of ventral surface approximately 15 mm from the tail tip using a radiant heat source provided by a light and thermalgesia algometer (Institute of Material Medica, Chinese Academy of Medical Sciences, Beijing, China). Each rat was held and gently restrained above the apparatus. A built-in timer was stopped automatically when the tail of the rat withdrew from the beam of light, and the result was displayed for viewing. Each rat was tested three times with an interval of 15 min and the mean of three times was the result of pain threshold.

#### 2.3.2. Transmission Electron Microscopy (TEM) to Observe the Myofibril Ultrastructure

When the rats were sacrificed, a piece of paraspinal muscle was harvested (1 × 1 × 1 mm^3^). The injured paraspinal muscle tissues were fixed in 5% glutaraldehyde for 1–3 h, washed in buffer, and then fixed in 1% osmic acid with pH adjusted to 7.2–7.4 at 4°C. The tissues were dehydrated with an ethanol gradient and were then placed in 100% acetone for 10 minutes. Tissue was then embedded in a mixture of 100% dehydrating agent and equivalent embedding medium (Epon 812) for 60 minutes, before they were finally placed in pure embedding medium overnight at 4°C. The structures of myofibers were analyzed with an electron microscope (Hitachi Ltd., Japan). Tissue sections of 80 nm were prepared and stained with acetic acid uranium saturated aqueous solution, followed by lead citrate. The tissues were examined for morphology changes such as arrangement rules of myofibrils, position of the sarcomere, sarcomere light band (I line), and dark zones (A line). Any abnormal Z-Lines, skeletal muscle cell membranes, nuclei, mitochondria, sarcoplasmic reticulum, and T-tubules were also observed.

#### 2.3.3. Serum Indicators

SP, 5-HT, TGF-*β*, IL-1*β*, IL-10, and TNF-*α* were tested by ELISA. Each blood sample was mixed with 90 *μ*L EDTA-Na_2_ and 120 *μ*L aprotinin and centrifuged (4°C, 2000 rpm) for 15 min to separate the serum for biochemistry and enzyme linked immunosorbent assay (ELISA).

### 2.4. Statistical Analysis

SPSS 21.0 statistical analysis software was used to analyze the data. All data are expressed as mean ± SD. The data of pain threshold was analyzed by nonparametric Kruskal-Wallis test, followed by multiple comparisons between treatment groups by LSD test. After the test of normal distribution and homogeneity, one-way ANOVA analysis was used for comparison of serological indicators between treatment groups, followed by post hoc multiple comparison. Statistical significance was set to *P* < 0.05, while highly statistical significance was set to *P* < 0.01.

## 3. Results

### 3.1. Ultrastructural Changes of Skeletal Muscles after Treatments

**Figure 1:**
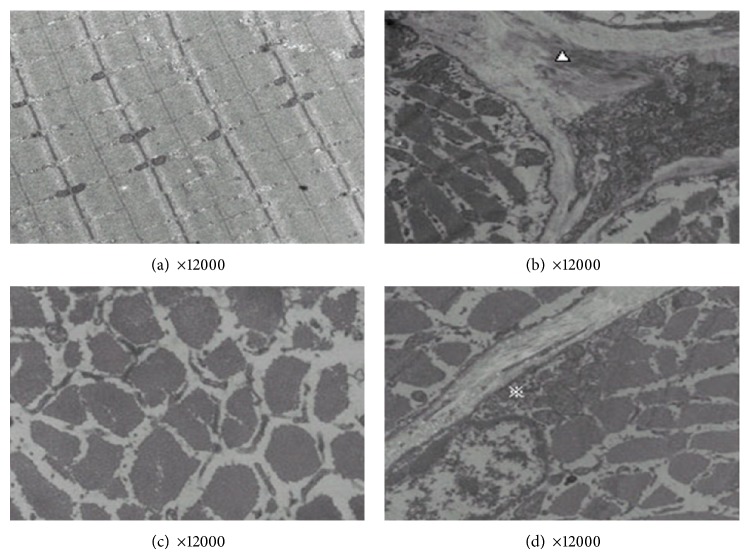
Representative ultrastructure changes of skeletal muscles after treatments in the following groups (*n* = 10 per group): (a) control, (b) model, (c) EA, and (d) AK.

In the control group, the structure of sarcomeres was well aligned, with orderly thick and thin filaments. A-bank, I-bank, H-bank, M-line, and Z-line were clear and regular. Around the junction of the A-bank and I-bank, there was a sarcoplasmic reticulum with obvious triads. The structure of mitochondria was clear with normal cristae. The sarcoplasmic reticulum distribution was regular with integrated muscle membrane (Figure 1(a)). In the model group, there was excessive empty space between the myofibrils. The structure of myofibrils and sarcomeres was disordered. A-bank, I-bank, H-bank, M-line, and Z-line were irregular or not present. Some thick or thin filaments were fractured. Swelling of mitochondria was observed. The number of irregularly arranged collagenous fibers was increased (Figure 1(b)). In the EA group, the empty space between the myofibrils was smaller than that in the model group. The number of fractured filaments was lower than that in the model group. Collagenous fibers were evident (Figure 1(c)). In the AK group, the empty space between the myofibrils was smaller than that in the model group. A small number of filaments were fractured. The structure of mitochondria was almost normal. Collagenous fibers were observed, but arrangement was orderly (Figure 1(d)).

### 3.2. Effects of Pain Threshold on Tail Flick Test

**Figure 2:**
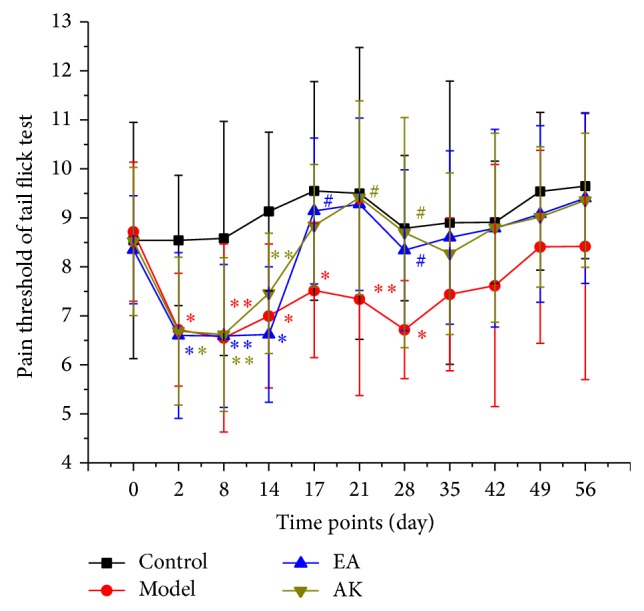
Effects of pain threshold on tail flick test in each group (*n* = 10 per group): control, model, EA, and AK. Compared with the control group, ^*^ *P* < 0.01, ^**^ *P* < 0.05. Compared with the model group, ^#^ *P* < 0.05.

The results of the pain threshold of tail flick test are shown in Figure 2. The pain threshold of the control group at different time points did not show obvious changes. The other three groups showed lower pain threshold from the second day after the operation. At 14 days, the day before performing treatment, compared with the control group, the model, EA, and AK group had a lower pain threshold (*P* < 0.01 or *P* < 0.05) and there were no statistical differences between the model, EA, and AK groups. After treatments, the pain threshold of the EA and AK group rose. From days 17 to 28, compared with the control group, the pain thresholds of the EA and AK groups were not statistically different. However, the pain threshold of the model group was significantly lower than the control group. On days 21 and 28, compared with the model group, the pain threshold of the AK group rose significantly (*P* < 0.05). From days 35 to 56, there was no significant difference between all four groups. However, Figure 1 shows a similar pain threshold between the control, EA, and AK group, which is higher than that of the model group.

### 3.3. Changes in IL-1*β*, IL-10, TNF-*α*, and TGF-*β* in the Peripheral Blood

**Figure 3:**
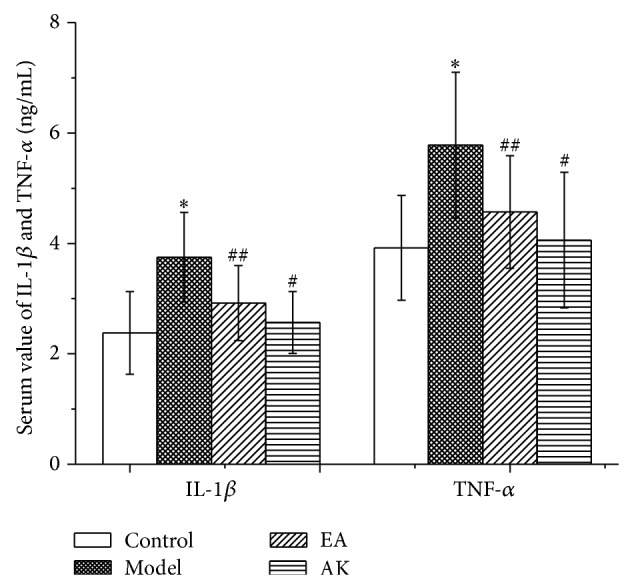
Effects of AK on IL-1*β* and TNF-*α* in peripheral blood in the following groups (*n* = 10 per group): control, model, EA, and AK. Compared with the control group, ^*^ *P* < 0.01; compared with the model group, ^#^ *P* < 0.01, ^##^ *P* < 0.05.

**Figure 4:**
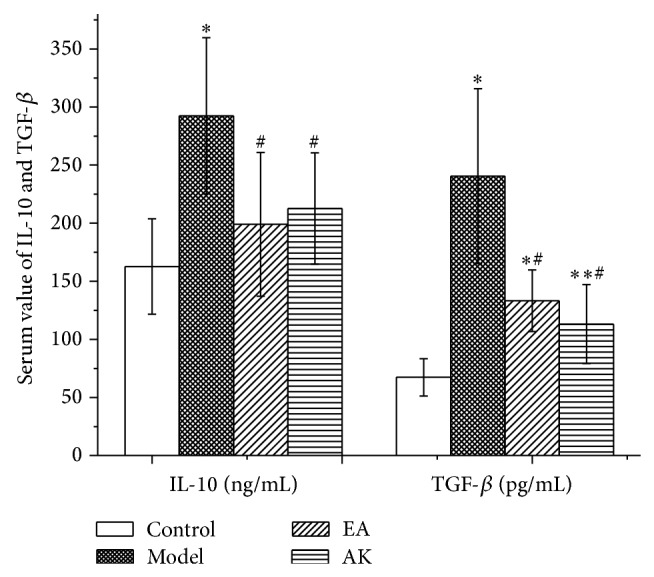
Effects of AK on IL-10 and TGF-*β* in peripheral blood in the following groups (*n* = 10 per group): control, model, EA, and AK. Compared with control group, ^*^ *P* < 0.01, ^**^ *P* < 0.05; compared with model group, ^#^ *P* < 0.01.

Fifty-six days after operation, IL-1*β* and TNF-*α* levels in the model group were significantly higher than that in the other three groups (*P* < 0.05 or *P* < 0.01). Compared with the control group, there was no significant difference in IL-1*β* and TNF-*α* in the EA and AK groups (*P* > 0.05). There was no significant difference in IL-1*β* and TNF-*α* between the EA and AK groups (*P* > 0.05) (Figure 3). The IL-10 level in the model group was significantly higher than that in the other three groups (*P* < 0.01). Compared with the control group, there was no significant difference in IL-10 in the EA and AK groups (*P* > 0.05). There were no significant differences in IL-10 among the EA and AK groups (*P* > 0.05). Compared with the control group, TGF-*β* in the model, EA, and AK groups were significantly higher (*P* < 0.01 or *P* < 0.05). Compared with the model group, TGF-*β* in the EA and AK groups were significantly lower (*P* < 0.01). There was no difference between the EA and AK groups (*P* > 0.05; Figure 4).

### 3.4. Changes in SP and 5-Hydroxytryptamine in the Peripheral Blood

**Figure 5:**
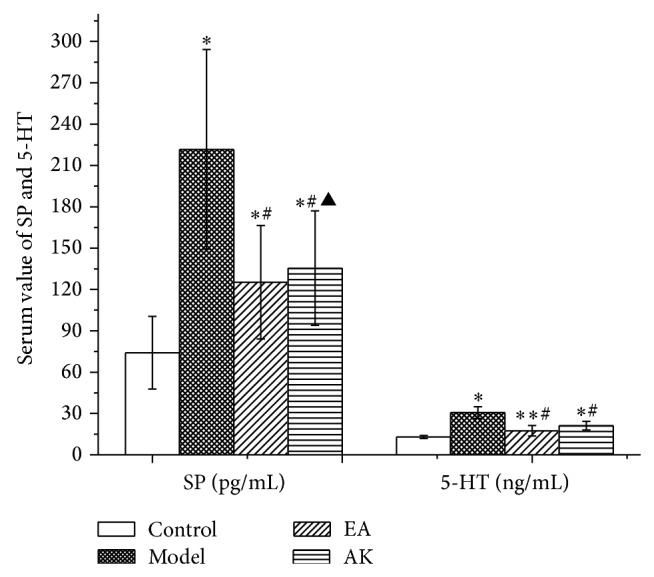
Effects of AK on SP and 5-HT in peripheral blood in the following groups (*n* = 10 per group): control, model, EA, and AK. Compared with the control group, ^*^ *P* < 0.01, ^**^ *P* < 0.05; compared with the model group, ^#^ *P* < 0.01; compared with the EA group, ^▲^ *P* < 0.05.

Fifty-six days after operation, compared with the control group, the model, EA, and AK groups had significantly higher SP levels in the peripheral blood (*P* < 0.05 or *P* < 0.01). Compared with the model group, the EA and AK groups had significantly lower SP levels in the peripheral blood (*P* < 0.01). There was no significant difference in SP among the EA and AK groups (*P* > 0.05). Compared with the control group, the model, EA, and AK groups had significantly higher 5-HT levels in the peripheral blood (*P* < 0.01). Compared with the model group, the EA and AK groups had significantly lower 5-HT levels in the peripheral blood (*P* < 0.01). The 5-HT of the AK group was higher than that of the EA group (*P* < 0.05; Figure 5).

## 4. Discussion

The term “tendon meridian” in traditional Chinese medicine is part of the meridian system, with functions of connecting limbs and regulating the movement of joints. Qi and blood in meridians nourish some tissues involved in movement, such as tendons, ligaments, fascia, skeletal muscles, and joint capsules. Some typical symptoms of tendon meridian disease are pain, numbness, hypertonicity, or stiffness of muscle or fascia, even motor dysfunction, which are possibly equivalent to chronic injury of the motor system. Third lumbar vertebrae transverse process syndrome is part of low back pain according to TCM theory, which is commonly because of qi and blood stagnation in the waist. Disordered blood circulation in the waist is caused by acute trauma, a chronic strain, or invasion of cold pathogens. The syndrome includes spontaneous unilateral regional pain near the third lumbar vertebrae transverse process or referred pain to the homolateral leg that is aggravated by bending down, with restricted range of waist motion, and the presence of hypersensitive nodules and local tenderness near the third lumbar vertebrae transverse process.

After acute or chronic injury, soft tissue adheres to the tip of the third lumbar vertebrae transverse process, which causes aseptic inflammation. Inflammation causes pathological changes such as edema, exudation, adhesion, or even continuous contracture in lesion tissues. Nociceptive terminals in muscle have a multitude of different receptors in their membranes, including matched receptors for molecules that are released from damaged tissue such as bradykinin, serotonin, and prostaglandins [10]. The continuous presence of these inflammatory mediators and other chemicals may be necessary for persistent pain conditions [10].

Cytokine TNF-*α* is a mediator of chronic pain produced by the onset of inflammation [11]. TNF-*α* can lead to NF-*κ*B-mediated transcription of IL-1, which mediates inflammatory hyperalgesia [12]. IL-1*β* is one of the most important members of the interleukin-1 family. When IL-1*β* binds to the IL-1 receptor, the activation of IL-1 receptors induces changes in synaptic strength and results in behavioral hyperalgesia [13]. Our results showed that serum TNF-*α* and IL-1*β* in the model rats remained at a higher level than that in the normal rats. This implies that the operation caused chronic inflammation. The increased cytokines confirmed the activation of immune cells and the production of sterile inflammation. The rat model of embedding absorbent gelatin is suitable for the observation of chronic inflammation and pain. After electroacupuncture and acupuncture knife treatments, the levels of serum TNF-*α* and IL-1*β* were significantly lower than those in model rats. Therefore, both the electroacupuncture and acupuncture knife treatments can reduce the release of cytokines. In addition, the effect of anti-inflammation is long term. Compared with EA rats, there were no significant differences in AK rats in both serum levels of TNF-*α* and IL-1*β*. However, the contents of TNF-*α* and IL-1*β* in AK rats were closer to those in normal rats, which suggests that acupuncture knife treatment is a more effective anti-inflammatory than electroacupuncture.

IL-10 is a powerful anti-inflammatory cytokine that can reduce the production of proinflammatory cytokines such as IL-1*β*, IL-6, TNF-*α*, and nerve growth factor. IL-10-producing monocytes and macrophages promote the resolution of transient inflammatory hyperalgesia. Impaired IL-10 production may represent a risk for developing chronic pain after inflammation [14]. Moreover, IL-10 gene therapy has been proven useful in neuropathic pain rats [15]. Our results showed that the serum IL-10 levels in model, EA, and AK rats were significantly higher than those in normal rats. However, compared with model rats, the serum levels in EA rats and AK rats were significantly lower. This result may prove the therapeutic effect of downregulation of proinflammatory cytokines by electroacupuncture and acupuncture knife treatments.

TGF-*β* has been recognized as a growth factor with multiple biological functions. TGF-*β* plays an important role in the regulation of immune cell function by limiting the production of inflammatory mediators, including interleukin-2 and tumor necrosis factor [16]. In chronic inflammation, TGF-*β* is expressed and promotes wound healing and fibrosis [17]. In addition, TGF-*β* promotes the expression of endogenous opioids, inhibiting the neuroimmune responses of glial cells and neurons in the spinal cord following peripheral injuries. Other undetermined mechanisms also contribute to its antiallodynic effect [18]. Our results showed that the serum levels of TGF-*β* in model, EA, and AK rats were significantly higher than those in normal rats, which implies that the peripheral injury tissue is under the fibroplasia stage. This was also shown by pathological observations. In addition, the content of TGF-*β* in EA and AK rats is significantly lower than that in model rats. This demonstrates that the level of TGF-*β* declined because of the lighter inflammatory response brought by EA and AK treatments.

Substance P (SP) is a neuropeptide released from nerve endings in many tissues, which is directly related with pain and plays an important role in inflammation [19]. Peripheral inflammation and noxious stimuli may trigger the release of SP [20], thus lowering pain thresholds and resulting in allodynia [21]. In the periphery, administration of 5-HT can increase the excitability of myelinated (A*δ* fibers) and unmyelinated afferents (C fibers) [22]. Previous study confirmed that the inhibition of 5-HT secretion from mast cells can diminish the sensitization of sensory nerve terminals [23]. Our results showed that, 56 days after operation, the concentration of SP and 5-HT in the blood of EA and AK rats was lower than that in model rats and higher than that in normal rats. Therefore, both EA and AK treatments contribute to relieving pain. This may be because of alleviation of local inflammatory responses. However, compared with AK rats, serum 5-HT of EA rats is significantly lower. This implies that EA treatment is more effective for alleviating pain.

## 5. Conclusions

In summary, we demonstrated that acupuncture knife therapy is able to mitigate pain by controlling the inflammatory response and is capable of restoring injury to soft tissue. However, the analgesic effect of electroacupuncture therapy is better than that of acupuncture knife therapy. This may be because of fewer treatment sessions for acupuncture knife therapy (two times) than electroacupuncture therapy (six times), accompanied by retaining the needle for 20 min. However, acupuncture knife therapy has a better trend for reducing inflammation. In addition, in spite of wider cutting edge of acupuncture knife than acupuncture needle, the puncture depth of acupuncture knife is shallower than acupuncture needle, that is, cutting in taut nodes and cords of muscle or fascia located under skin without arteries, veins, or nerves which can lead to irreversible damages. The safety of acupuncture knife is credible.
